# Low-loss and compact arbitrary-order silicon mode converter based on hybrid shape optimization

**DOI:** 10.1515/nanoph-2024-0301

**Published:** 2024-08-30

**Authors:** Junpeng Liao, Dongmei Huang, Yegang Lu, Yan Li, Ye Tian

**Affiliations:** 47862 Ningbo University, Ningbo, China; Hong Kong Polytechnic University, Hong Kong SAR, China

**Keywords:** integrated photonics, inverse design, mode-division multiplexing

## Abstract

Mode converters (MCs) play an essential role in mode-division multiplexing (MDM) systems. Numerous schemes have been developed on the silicon-on-insulator (SOI) platform, yet most of them focus solely on the conversion of fundamental mode to one or two specific higher-order modes. In this study, we introduce a hybrid shape optimization (HSO) method that combines particle swarm optimization (PSO) with adjoint methods to optimize the shape of the S-bend waveguide, facilitating the design of arbitrary-order MCs featuring compactness and high performance. Our approach was validated by designing a series of 13 μm-long MCs, enabling efficient conversion between various TE modes, ranging from TE_0_ to TE_3_. These devices can be fabricated in a single lithography step and exhibit robust fabrication tolerances. Experiment results indicate that these converters achieve low insertion losses under 1 dB and crosstalks below −15 dB across bandwidths of 80 nm (TE_0_–TE_1_), 62 nm (TE_0_–TE_2_), 70 nm (TE_0_–TE_3_), 80 nm (TE_1_–TE_2_), 55 nm (TE_1_–TE_3_), and 75 nm (TE_2_–TE_3_). This advancement paves the way for flexible mode conversion, significantly enhancing the versatility of on-chip MDM technologies.

## 1 Introduction

To accommodate the increasing demands for data transmission capacity, on-chip optical interconnects are regarded as a promising solution due to their broad bandwidth, high speed, and low power consumption. On-chip mode-division multiplexing (MDM) technology enables the transmission of optical signals by simultaneously exploiting each eigenmode supported by multimode waveguides as an independent channel, offering a new dimension to significantly enhance the transmission capacity of photonic integrated circuits (PICs) [1], [2]. The implementation of MDM technology involves various multimode devices, such as mode (de)multiplexers [(De)MUXers] [3], [4], mode converters (MCs) [5], [6], multimode waveguide bends (MWBs) [7], etc. Among them, MCs are crucial for enabling conversions between different modes. Recent advancements have seen countless MC designs on silicon-on-insulator (SOI) platforms, including asymmetric directional couplers (ADCs) [8], [9], Mach–Zehnder interferometers (MZIs) [10], Y-junctions [11], adiabatic tapers [12], and Bragg gratings [13]. However, these designs generally have a large footprint, which is unfavorable to the high integration density of on-chip PICs.

Subwavelength grating (SWG) structures have emerged as a potential solution to facilitate high-performance MCs within a compact size [14]–[17]. By engineering optical properties through SWGs, the device footprint can be reduced to several square microns while maintaining losses below 1 dB [15], [16]. Recently, Guo *et al*. experimentally demonstrated a flexible and high-performance MC by optimizing the SWG region, achieving efficient arbitrary conversions spanning TE_0_ to TE_3_ modes [17]. Despite these advancements, SWG-based devices are constrained by strict fabrication precision and limited operation bandwidth.

Recently, inverse design methods have been increasingly utilized to engineer devices with flexible functions within compact footprint [18]–[21]. Various MC designs have been developed by applying these methods to design functional regions with a pixelated structure [22]–[25]. Wang *et al*. employed a direct binary search (DBS) algorithm to achieve a TE_0_ to TE_1_ conversion with an ultra-compact length of 2 μm and a loss below 2.2 dB over a 40 nm bandwidth [24]. Topological Optimization (TO) can achieve more efficient device design through gradient updating strategies [26], [27]. TE_0_ to TE_1_ mode conversion has been demonstrated using this method [28]–[30]. The inverse design method can also be extended to realize arbitrary mode conversion. Dou *et al*. employed an adaptive genetic algorithm (AGA) to optimize several fully etched rectangular nanoholes on a multimode silicon waveguide, theoretically demonstrating arbitrary-order conversion within an ultra-compact size of 1.8 × 2.2 μm^2^ [31]. However, the loss within a 300 nm bandwidth was as high as 3 dB. Additionally, these pixelated structures require high-precision fabrication processes, as well as consideration of robustness to over/under etching. The inverse design of the device shape through various optimization methods is an alternative way to achieve efficient mode conversion [32]. Li *et al*. used a gradient-based method of moving asymptotes, and silicon waveguides with a length of 6 μm are reshaped to achieve TE_0_ to TE_1_ and TE_1_ to TE_2_ conversions [33]. Theoretical analysis shows low losses of less than 0.1 dB and 0.25 dB across a 60 nm bandwidth for these converters. The shape-optimized MCs, as an alternative, are preferred for optimizing structures with relatively larger sizes while maintaining comparable computational complexity. By avoiding nanoscale holes within the structure, shape-optimized MCs have excellent robustness to fabrication errors.

Most existing works demonstrate only the conversion from the fundamental mode TE_0_ to lower-order modes such as TE_1_ or TE_2_, with limited attempts for higher-order modes. Higher-order modes exhibit weaker optical field confinement, requiring more space for efficient mode propagation and conversion. Consequently, the device size must be increased for higher-order mode conversions, as noted in references [14], [17], [24]. This significantly elevates the computational complexity and time, particularly for algorithms such as DBS, where the computational cost grows exponentially. Furthermore, direct conversion between higher-order modes has some potential for applications like mode add/drop multiplexers (MADM) and higher-order mode filters [34], [35]. Whereas references [12]–[14] employ cascading strategies to achieve this, which leads to increased losses and footprint. Therefore, realizing a high-performance arbitrary-order mode converter in a cost-effective manner remains a significant challenge.

In this paper, we propose and experimentally demonstrate an arbitrary-order MC with low loss and compact size. The converter is realized by the inverse design of the S-shaped bend with a fixed length of 10 μm through a novel hybrid shape optimization (HSO) method, which incorporates a two-step optimization process. Initially, PSO is applied to efficiently refine the geometry of two Bezier curves that delineate the shape of the MC. Subsequently, the adjoint method is utilized to fine-tune the boundary shapes, significantly enhancing the conversion efficiency. By combining these two steps, the local optimization issue can be mitigated. To verify its functionality, we developed six MCs, covering conversions from TE_0_ to TE_1_, TE_0_ to TE_2_, TE_0_ to TE_3_, TE_1_ to TE_2_, TE_1_ to TE_3_, and TE_2_ to TE_3_. Simulations indicate that these converters achieve insertion losses (IL) below 0.2 dB and crosstalk (CT) less than −20 dB at a center wavelength of 1,550 nm. The calculated operation bandwidths with ILs below 1 dB and CTs under −15 dB are 130 nm, 100 nm, 100 nm, 170 nm, 65 nm, and 90 nm, respectively. The devices are based on a standard 220 nm SOI platform with robust fabrication tolerances and can be fabricated with the mature 193 nm deep ultraviolet lithography (DUV) through the Multi-Project Wafer (MPW) process for large-volume production. Experimental results revealed ILs under 1.2 dB across a wavelength range from 1,500 to 1,580 nm for all the converters, while the bandwidths for CTs below −15 dB varied from 55 nm to 80 nm. Overall, the proposed MCs outperform the previous designs considering the footprint, loss, crosstalk, and operation bandwidth.

## 2 Design and simulations

**Figure 1:**
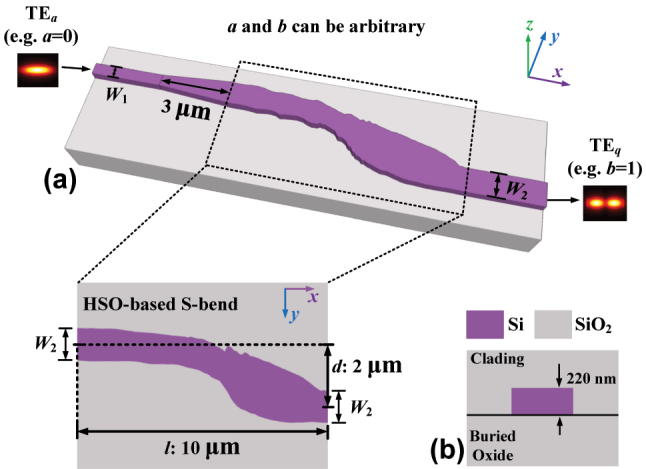
The arbitrary-order MC based on HSO. (a) The schematic diagram of the proposed arbitrary-order MC. (b) Cross section of the waveguide.

Figure 1(a) illustrates the three-dimensional (3D) schematic of the proposed arbitrary-order MC. The converter comprises an input waveguide with a width of *W*_1_, an output waveguide with a width of *W*_2_, and an S-bend waveguide obtained through HSO. The S-bend structure is chosen as the MC because it can disrupt the symmetry of the input modes, facilitating the interconversion between two modes with different symmetries [36]. The widths of *W*_1_ and *W*_2_ are determined based on the desired mode order and the interface of the device with the rest of the test circuit. While the length *l* and latitude distance *d* of the S-bend are set as 10 μm and 2 μm, respectively. Additionally, a tapered waveguide with a length of 3 μm is introduced between the S-bend and the input waveguide to enlarge the input mode spot size and improve the mode conversion efficiency. The proposed devices are designed on the SOI platform, featuring a 220 nm thick core silicon layer and 1 μm top oxide cladding. The cross-sectional view of this structure is depicted in Figure 1(b).

**Figure 2:**
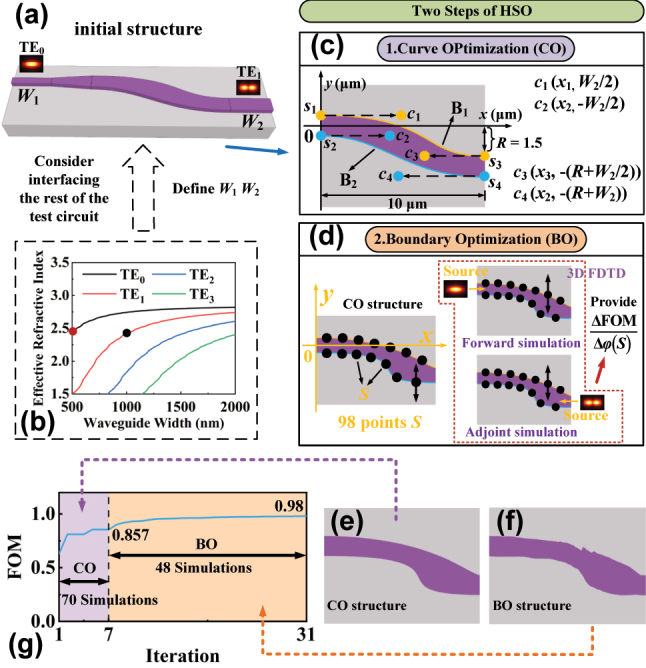
The process of designing the MC using the HSO. (a) Initial structure of the converter. (b) Effective refractive index as a function of width variation for different TE modes at the 1,550 nm. (c) The curve optimization step. (d) The boundary optimization step. (e) S-bend after curve optimization. (f) S-bend after boundary optimization. (g) The evolution of the FOM.

To illustrate our process of designing MCs using the HSO method in detail, we present the design of a TE_0_ to TE_1_ MC as an example. As shown in Figure 2(a), the initial structure of the MC is based on an S-bend defined by Bezier curves. Considering the connection of the device to the ADC-based mode (De)MUXers used for testing in the subsequent test circuit, we calculated the effective refractive index of the TE modes at 1,550 nm varying with the waveguide width using the Finite-Difference Eigenmode (FDE) method, as shown in Figure 2(b). Based on the effective refractive index matching, the input width of the converter *W*_1_ was determined to be 500 nm to support the TE_0_ mode, and the output width *W*_2_ was determined to be 1 μm to support the TE_1_ mode. The proposed HSO method consists of two essential steps: curve optimization (CO) and boundary optimization (BO). The CO step aims to rapidly obtain the shape of the MC by optimizing two Bezier curves using the PSO technique, thereby providing the initial conditions for the BO step. The BO step further fine-tunes the device boundaries, which is performed using the adjoint shape optimization technique, resulting in an MC with improved performance. Adjoint shape optimization tends to converge to poorly performing locally optimal solutions that strongly depend on the initial conditions [37]. HSO mitigates this problem by combining a CO step with a BO step, and the device structure can be precisely optimized to achieve superior performance.

In the CO step, we use two cubic Bezier curves, B_1_(*t*) and B_2_(*t*) (0 ≤ *t* ≤ 1), to construct the S-bend structure of the MC, as shown in Figure 2(c). These curves are labeled in yellow and blue, respectively, which can be written as:

(1) $$B_{1}\left(t\right)=s_{1}{\left(1-t\right)}^{3}+3c_{1}t{\left(1-t\right)}^{2}+3c_{3}t^{2}\left(1-t\right)+s_{3}t^{3}$$

(2) $$B_{2}\left(t\right)=s_{2}{\left(1-t\right)}^{3}+3c_{2}t{\left(1-t\right)}^{2}+3c_{4}t^{2}\left(1-t\right)+s_{4}t^{3}$$

The Bezier curves B_1_(*t*) and B_2_(*t*) are defined by their starting points (*s*_1_ and *s*_2_), ending points (*s*_3_ and *s*_4_), and controlling points (*c*_1_, *c*_2_, *c*_3_, and *c*_4_). The controlling points *c*_1_ and *c*_3_ influence the shape of B_1_(*t*), while *c*_2_ and *c*_4_ control the shape of B_2_(*t*). By adjusting the coordinates of these controlling points, the shape of the Bezier curves can be easily modified, providing convenient and intuitive control of the shape of the MC. To ensure a smooth transition between the S-bend and the input/output waveguide, certain relationships between the coordinates of the controlling points are maintained. Specifically, the *y*-coordinates of *c*_1_ and *c*_2_ align with those of *s*_1_ and *s*_2_, respectively. Similarly, the *y*-coordinates of *c*_3_ and *c*_4_ align with *s*_3_ and *s*_4_, respectively. In this case, refer to the origin of the coordinates, the coordinates of *s*_1_, *s*_2_, *s*_3_, and *s*_4_ are set to (0, *W*_2_/2), (0, −*W*_2_/2), (*l*, −(*d* − *W*_2_/2)), and (*l*, −(*d* + *W*_2_/2)), respectively. The coordinates of *c*_1_ and *c*_3_ are set to (*x*_1_, *W*_2_/2) and (*x*_2_, −(*d* − *W*_2_/2)), respectively, while the coordinates of *c*_2_ and *c*_4_ are set to (*x*_3_, −*W*_2_/2) and (*x*_4_, −(*d* + *W*_2_/2)), respectively. The CO step focuses on optimizing parameters *x*_1_, *x*_2_, *x*_3_, and *x*_4_, which correspond to the *x*-coordinates of the four controlling points. By adjusting these parameters, the shape of the S-bend can be optimized to achieve the desired performance.

We use PSO to optimize these parameters. PSO has been widely used in the field of inverse design due to its fast convergence and effectiveness in solving multi-parameter optimization problems [38], [39]. PSO has advantages in terms of algorithmic simplicity compared to other heuristic optimization methods. Moreover, PSO exhibits better performance than other heuristic optimization methods with increasing problem dimensionality [40], [41]. The three-dimensional finite-difference time-domain (3D FDTD) method is utilized for optical propagation simulation and analysis. To ensure a comprehensive search for the optimal parameters, a wide range of parameter values is set for optimization: *x*_1_, *x*_2_, *x*_3_, *x*_4_ ∈ [0, 10 μm]. The optimization objective is quantified by the figure of merit (FOM), defined as the average transmittance of the TE_1_ mode at the output over the designed bandwidth,

(3) $$FOM=\frac{1}{N}\overset{\lambda_{\max}}{\underset{\lambda_{\min}}{\sum }}\left(T_{\text{out}}\left(\lambda \right)\right)$$

where *T*_out_(*λ*) is the transmittance of the output TE_1_ mode at wavelength *λ*. *λ*_min_ and *λ*_max_ represent the minimum and maximum wavelengths of the target bandwidth, respectively. *N* is the number of samples we select within the range of *λ*_min_ and *λ*_max_. In PSO, the positions of the particles represent the values of *x*_1_, *x*_2_, *x*_3_, and *x*_4_, while the velocities of the particles correspond to the variation ranges of these parameters. The positions and velocities of the particles are updated iteratively based on the following equations [42]:

(4) $$\begin{array}{c} v_{n}=\omega v_{n-1}+r_{1}\eta_{1}\left(p_{\text{best,}n-1}-z_{n-1}\right)\\ +r_{2}\eta_{2}\left(g_{\text{best,}n-1}-z_{n-1}\right) \end{array}$$

(5) $$z_{n}=z_{n-1}+v_{n}$$

where *z* and *v* represent the position and velocity of the particle, respectively. *n* denotes the iteration. *p*_best_ represents the best position achieved by the particle so far, and *g*_best_ represents the best position achieved by any particle in the entire swarm. The coefficients *ω*, *r*_1_, and *r*_2_ control the impact of the previous velocity, the particle’s personal best, and the swarm’s global best on the update. *η*_1_ and *η*_2_ are random coefficients between 0 and 1. By updating the velocities and positions of the particles in each iteration, the PSO algorithm explores the solution space and converges toward the optimal values for the parameters *x*_1_, *x*_2_, *x*_3_, and *x*_4_. In our design, the number of particles in each iteration is fixed to 10, with each particle representing a different set of parameters, and the total iterations fixed to 7. For the TE_0_ to TE_1_ MC, the FOM reaches 0.857 after the CO step, with *x*_1_, *x*_2_, *x*_3_, and *x*_4_ determined to be 6.95, 10, 9.285, and 2.689, respectively.

In the BO step, the performance is further improved by fine-tuning the boundaries of the MC and raising the FOM value. To perform the BO, a total of 98 discrete boundary optimization points, denoted as *S*, are uniformly inserted along the two boundaries of the S-bend obtained through CO, as shown in Figure 2(d). The boundary shape of the S-bend is optimized by adjusting the coordinates of these points *S*. By modifying the boundary shape of the waveguide, refractive index perturbations are introduced, affecting the transmission of the corresponding modes within the device to achieve the design target. To enhance the optimization efficiency, the adjoint method is introduced in the BO step. By employing one forward simulation and one adjoint simulation, the adjoint method provides shape gradient information regardless of the number of design parameters, significantly reducing the simulation frequency and minimizing the computational cost [43], [44]. The partial derivative of the FOM at each point on the structural boundary can be found using the adjoint method, which can be expressed as [45]:

(6) $$\begin{array}{cc} \frac{\partial FOM}{\partial \varphi \left(S\right)} & =2Re\left[\left(\varepsilon_{2}-\varepsilon_{1}\right)E_{\|}\left(S\right)\cdot E_{\|}^{\text{adj}}\left(S\right)\right.\\ & \;\left.+\left(\frac{1}{\varepsilon_{1}}-\frac{1}{\varepsilon_{2}}\right)D_{⊥}\left(S\right)\cdot D_{⊥}^{\text{adj}}\left(S\right)\right] \end{array}$$

where *ɛ*_1_ and *ɛ*_2_ are the permittivity of the cladding material (SiO_2_) and Si material, respectively. *E*_‖_(*S*) and $E_{\|}^{\text{adj}}$ (*S*) (*D*_⊥_(*S*) and $D_{⊥}^{\text{adj}}$ (*S*)) are the tangential (normal) components of the electric field (electric displacement) at the boundaries obtained from forward and adjoint simulations, respectively. ∂*φ*(*S*) is the size of the deformation at points *S* along the boundary.

Therefore, firstly, we perform forward and adjoint simulations using 3D FDTD to collect the information on the electric field and electric displacement, as shown in Figure 2(d). Subsequently, the gradient ∂FOM/∂*φ*(*s*) was calculated with Python. The gradient descent algorithm is then employed to predict the coordinates of the points *S* that can improve the FOM. After that, the coordinates are adjusted and points *S* are interconnected using spline interpolation to construct the new boundary curves. To avoid generating overly sharp structures, numerical constraints have been imposed on the *y*-axis coordinate of the optimization point *S*, limiting its variation range to within ±0.2 μm. The optimization process is repeated until the difference between the FOM values of two adjacent iterations of the device is less than 10^−3^. The S-bend optimized through the CO and BO steps are depicted in Figure 2(e) and (f), respectively. Figure 2(g) presents the evolution of the FOM throughout the entire design process, where it can be observed that after 7 iterations of the CO step, the FOM gradually converges to 0.857. With the assistance of the BO step, the FOM further improves to 0.98 after 24 iterations. This demonstrates that the HSO method significantly enhances the transmittance of the TE_1_ mode. Therefore, we can quickly implement mode conversion of arbitrary order using HSO by simply modifying the appropriate input/output widths and determining the optimization objective.

**Figure 3:**
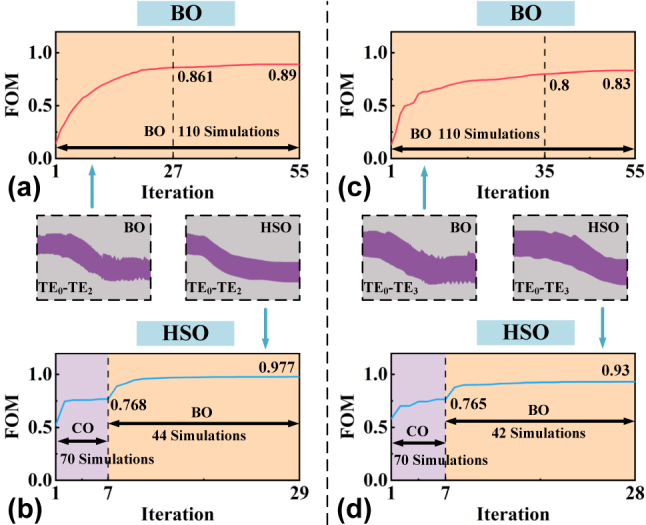
The FOM evolution for TE_0_ to TE_2_ converter designed using (a) BO solely and (b) with HSO method. The FOM evolution for TE_0_ to TE_3_ converter designed (c) using BO solely and (d) with HSO. Inset: the schematic diagram of the final optimized S-bends.

Meanwhile, to further demonstrate the necessity of the CO step, we optimized two sets of converters (TE_0_ to TE_2_ and TE_0_ to TE_3_) using both the BO and HSO methods, starting from the same initial structure, and compared the FOM evolutions, as depicted in Figure 3. As shown in Figure 3(a) and (c), using the BO method alone, the FOMs rise rapidly in the first 20 iterations and converge to 0.89/0.83 by the end. In comparison, assisted by the HSO method, the FOMs rise rapidly in the first 10 iterations and eventually converge to 0.977/0.93, as shown in Figure 3(b) and (d). CO provides a favorable starting point for BO, effectively mitigating the issue of local optimization. The insets present the schematic diagram of the finally optimized S-bends, and it is evident that the HSO-based S-bends exhibit smoother boundaries, making them more favorable for fabrication. Since heuristic optimization algorithms rely on random testing of a large number of different parameters, a large number of electromagnetic simulations are required in the design process compared with gradient algorithms [46]. In Figure 3, we marked the number of 3D FDTD simulations used in the two designs. It can be found that there is no significant difference in the number of simulations using the HSO and BO methods, which also avoids the shortcomings of PSO to a certain extent. The HSO method is implemented on a workstation equipped with a 20-core CPU (Intel Xeon W-2255), where the design of each MC is completed in about 12 h. With a higher configuration workstation, the design time is expected to be reduced to within 10 h.

**Figure 4:**
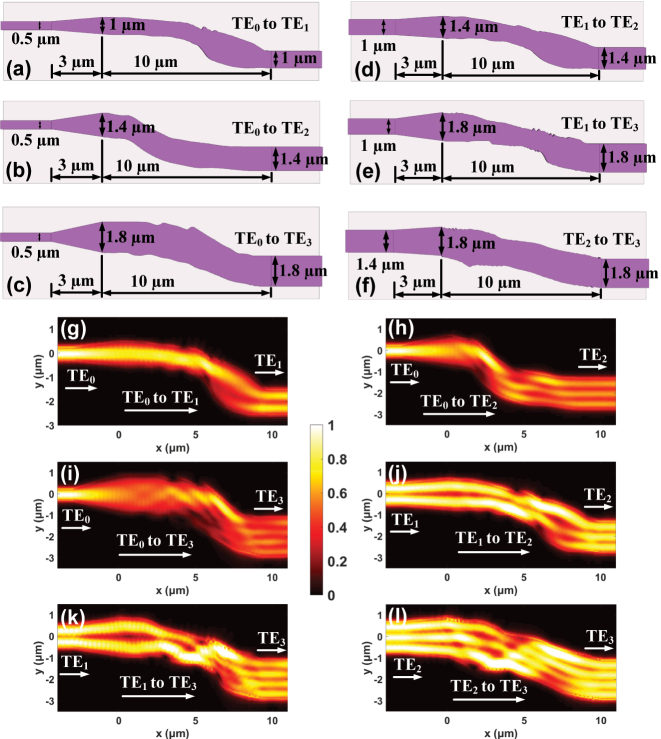
The six MCs based on HSO. (a)–(f) The schematic diagram of the designed MCs. (g)–(l) Simulated mode conversions within these converters at 1,550 nm.

**Figure 5:**
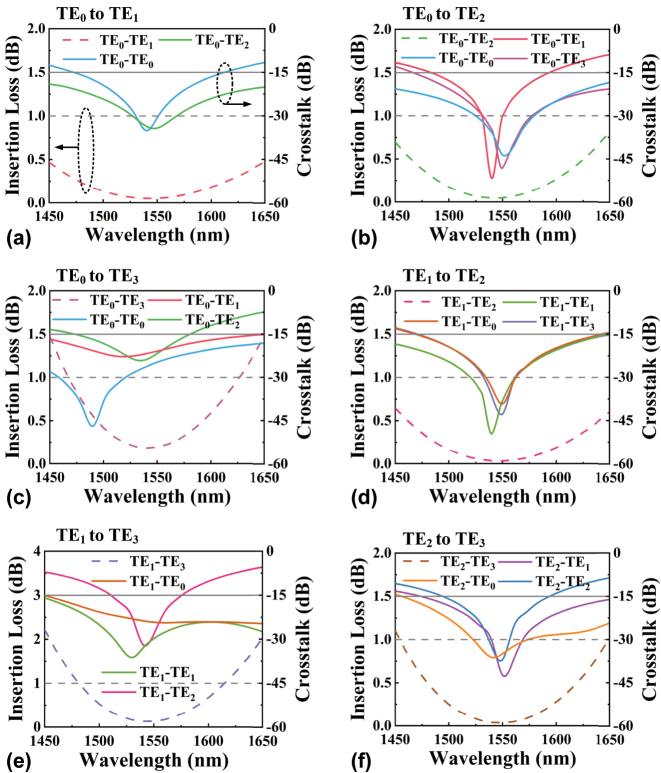
The calculated ILs and CTs for (a) TE_0_ to TE_1_, (b) TE_0_ to TE_2_, (c) TE_0_ to TE_3_, (d) TE_1_ to TE_2_, (e) TE_1_ to TE_3_, and (f) TE_2_ to TE_3_ converters over a wavelength range from 1,450 to 1,650 nm. Herein, the dashed line refers to the axis on the left side, and the solid line refers to the axis on the right side.

To showcase our capability for arbitrary-order mode conversion using HSO, we designed six converters, each for TE_0_ to TE_1_, TE_0_ to TE_2_, TE_0_ to TE_3_, TE_1_ to TE_2_, TE_1_ to TE_3_, and TE_2_ to TE_3_ conversion. The schematic geometries of the six mode converters designed are presented in Figure 4(a)–(f), each with a length of 13 μm. The simulated electric field distributions at 1,550 nm for all devices are illustrated in Figure 4(g)–(l), showing that all the input modes are gradually transformed into the target modes via optimized S-bend structures. The calculated insertion losses (ILs) and crosstalks (CTs) across a broad bandwidth from 1,450 to 1,650 nm for all the converters are shown in Figure 5(a)–(f). Here, the IL and CT are defined as

(7) $$IL=-10{log}_{10}\left(\frac{T_{\text{target}}}{T_{\text{int}}}\right)$$

(8) $$CT=10{log}_{10}\left(\frac{T_{\text{other}}}{T_{\text{int}}}\right)$$

where *T*_int_, *T*_target_, and *T*_other_ are the transmittance of the input mode, the target mode, and the nontarget modes, respectively. For TE_0_ to TE_1_, TE_0_ to TE_2_, TE_0_ to TE_3_, TE_1_ to TE_2_, TE_1_ to TE_3_, and TE_2_ to TE_3_ converters, the ILs at 1,550 nm are remarkably low at 0.051 dB, 0.048 dB, 0.195 dB, 0.032 dB, 0.128 dB, and 0.033 dB, while the CTs are below −34 dB, −43 dB, −20 dB, −39 dB, −24 dB, and −34 dB, respectively. To evaluate the broadband performance of the device, we define the operation bandwidth as the range of wavelengths over which the ILs remain below 1 dB and the CTs are less than −15 dB simultaneously. Consequently, the bandwidths achieved for TE_0_ to TE_1_, TE_0_ to TE_2_, TE_0_ to TE_3_, TE_1_ to TE_2_, TE_1_ to TE_3_, and TE_2_ to TE_3_ converters are 130 nm (1,480–1,610 nm), 100 nm (1,490–1,590 nm), 100 nm (1,480–1,580 nm), 170 nm (1,470–1,640 nm), 65 nm (1,510–1,575 nm), and 90 nm (1,500–1,590 nm). These MCs exhibit superior performance with low loss, low crosstalk, and broad operation bandwidth, surpassing most of the reported works [13], [22], [24].

**Figure 6:**
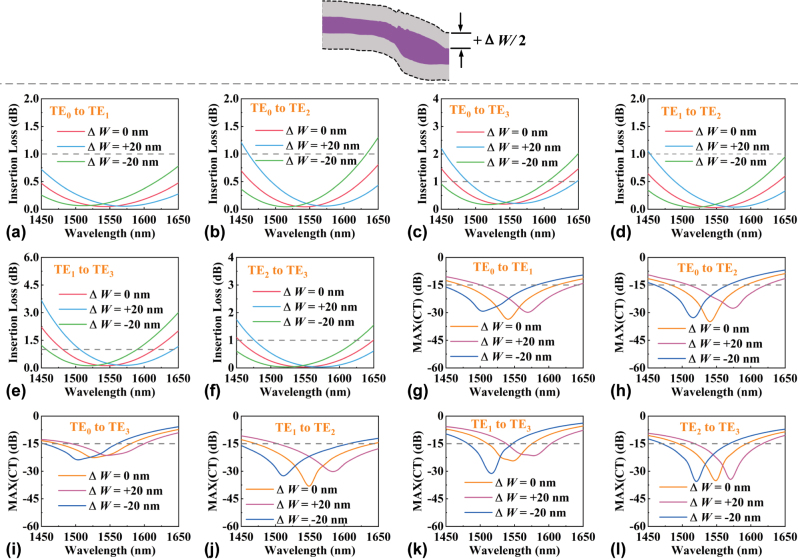
Fabrication error analysis when changing waveguide width. (a)–(f) Calculated ILs with Δ*W* varying ±20 nm over the whole band. (g)–(l) The maximum CT with Δ*W* varying ±20 nm over the band.

A tolerance analysis was conducted to investigate the impact of fabrication errors on device performance. This analysis involved calculating the IL and CT for the MCs while introducing the width variations (Δ*W*). Figure 6(a)–(f) provide an in-depth examination of the ILs for the six converters over the wavelength range from 1,450 to 1,650 nm with Δ*W* varying by ±20 nm. Additionally, the maximum CT for all nontarget modes over the band is shown in Figure 6(g)–(l). It is observed that the center wavelength experiences a blueshift when the width increases. However, for ILs of below 1 dB and CTs of less than −15 dB, all the converters still exhibit operation bandwidths of 130 nm, 100 nm, 80 nm, 140 nm, 50 nm, and 90 nm, respectively. This indicates that our proposed MCs have robust tolerance to fabrication errors.

## 3 Fabrication and experiment

**Figure 7:**
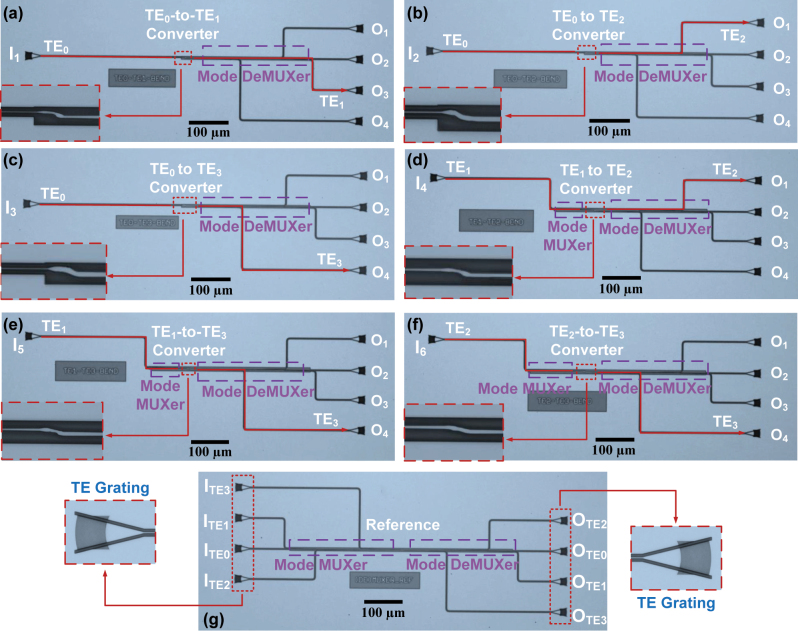
Microscope images of the on-chip test setup for (a) TE_0_ to TE_1_, (b) TE_0_ to TE_2_, (c) TE_0_ to TE_3_, (d) TE_1_ to TE_2_, (e) TE_1_ to TE_3_, and (f) TE_2_ to TE_3_ converters, respectively. Inset: enlarged microscopic images of the corresponding fabricated MCs. (g) Microscopic image of the four-channel mode MUXer and DeMUXer connected back to back.

The proposed MCs were fabricated through a commercial MPW run on an SOI wafer with a 220 nm top silicon layer and a 2 μm buried oxide layer. The device patterns were defined using 193 nm DUV photolithography while the silicon layers were etched using inductively coupled plasma (ICP). A silica upper cladding was subsequently deposited on the structure by a plasma-enhanced chemical vapor deposition (PECVD) process. Figure 7(a)–(f) show the microscope images of the on-chip test setups for TE_0_ to TE_1_, TE_0_ to TE_2_, TE_0_ to TE_3_, TE_1_ to TE_2_, TE_1_ to TE_3_, and TE_2_ to TE_3_ converters, respectively, with insets showing the zoom-in views of each converter. To enable the multiplexing and demultiplexing of all the desired modes, mode multiplexers (MUXers) and demultiplexers (DeMUXers) based on ADC structures were implemented [47]. For a TE_0_ input, light is directly launched into the bus waveguide connected to the S-bend. For high-order mode inputs, the light is injected into a specific port and couples to the corresponding high-order mode within the bus waveguide. After the mode conversion through the S-bend structure, the output modes are converted into TE_0_ mode by the DeMUXers, exiting from the corresponding port. Besides, two identical (De)MUXers were connected back-to-back and measured for the normalization of the transmittance, as shown in Figure 7(g). All ports are connected to TE-type grating couplers (GCs) with a minimum loss of 5 dB/facet for light coupling between the chip and fibers. It should be mentioned that the GCs have a limited bandwidth. Since different tilt angles of the optical fiber result in different central wavelengths, we obtain relatively broader transmission spectra by adjusting the tilt angles. During our experiment, a broadband amplified spontaneous emission (ASE) source served as the input, and an optical spectrum analyzer (OSA, AQ6317B) was employed to monitor the transmission spectra. However, due to the limited bandwidth of ASE and the GCs, the device performance was analyzed over a bandwidth from 1,500 to 1,580 nm.

**Figure 8:**
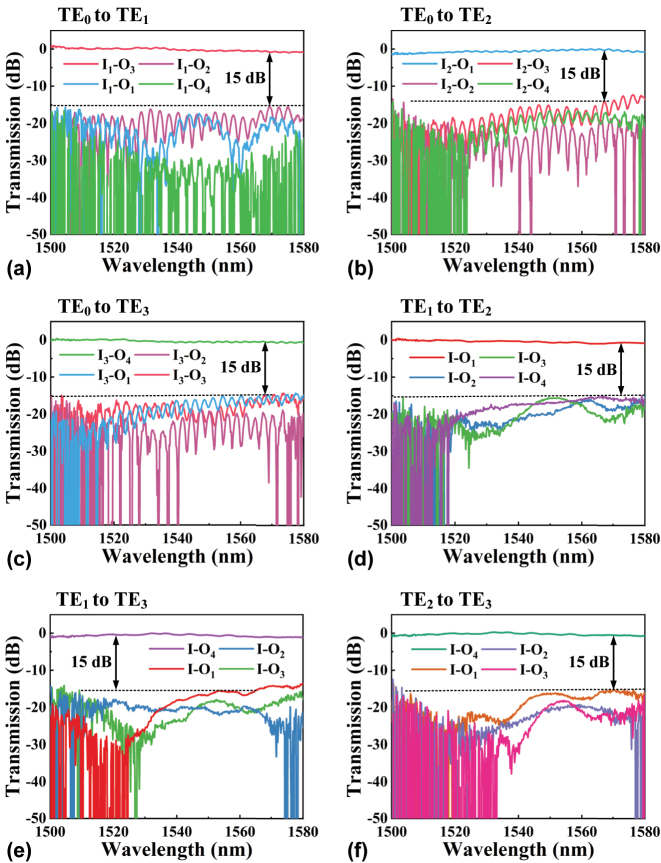
Measured transmission spectra for fabricated, (a) TE_0_ to TE_1_, (b) TE_0_ to TE_2_, (c) TE_0_ to TE_3_, (d) TE_1_ to TE_2_, (e) TE_1_ to TE_3_, and (f) TE_2_ to TE_3_ converters.

The losses from the (De)MUXers along with the GCs are deducted from the measured spectra. The normalized transmission spectra of the six converters are displayed in Figure 8(a)–(f). Over the wavelength range of 1,500–1,580 nm, the ILs for the TE_0_ to TE_1_, TE_0_ to TE_2_, TE_0_ to TE_3_, TE_1_ to TE_2_, TE_1_ to TE_3_, and TE_2_ to TE_3_ converters are below 0.8 dB, 1.2 dB, 0.8 dB, 0.9 dB, 1.2 dB, and 0.8 dB, respectively, while the CTs are below −15 dB, −12 dB, −14 dB, −15 dB, −13 dB, and −12 dB, respectively. Furthermore, for IL of less than 1 dB and CT of below −15 dB, the six converters exhibit bandwidths of 80 nm (1,500–1,580 nm), 62 nm (1,505–1,567 nm), 70 nm (1,500–1,570 nm), 80 nm (1,500–1,580 nm), 55 nm (1,510–1,565 nm), and 75 nm (1,505–1,580 nm). It is worth noting that slight differences between the measured and the simulated results have been observed. These discrepancies can be attributed to random deviations in waveguide sidewall etch roughness. Additionally, interference patterns observed in the measured transmission spectra are likely caused by reflections from the GCs and the ADCs.

Table 1 provides a comprehensive comparison between previously demonstrated MCs on the SOI platform and the MCs presented in this work. Our devices achieve flexible conversions spanning TE_0_ to TE_3_ modes. They have a more compact footprint compared to conventional designs such as ADC and MZI. Additionally, our MCs exhibit advantages in terms of loss, crosstalk, and bandwidth compared to other inverse designs.

**Table 1:** Comparison of demonstrated MCs on SOI platform.^a^

Method	Function	Length (μm)	IL (dB)		CT (dB)		Bandwidth (nm)	
			Sim.	Exp.	Sim.	Exp.	Sim.	Exp.
ADC [8]	TE_0_ to TE_1_	15.5	–	<1	–	<–15	–	30
	TE_0_ to TE_2_	21.3	–	<0.8	–	<–17	–	30
	TE_0_ to TE_3_	17.6	–	<1	–	<–13	–	30
MZI [10]	TE_0_ to TE_1_	60	–	<1	–	<–24	–	35
	TE_1_ to TE_0_	60	–	<1	–	<–24	–	35
Bragg grating [13]	TE_0_ to TE_1_	65.3	<0.5	–	<–21	–	25	–
	TE_0_ to TE_2_	60	<0.5	–	<–23.5	–	30	–
Polygonal SWG [16]	TE_0_ to TE_1_	4.44	<0.2	<1	<–15	<–7.2	100	50
	TE_0_ to TE_2_	5.89	<0.24	<1	<–15	<–10.3	100	50
Bricked SWG [15]	TE_0_ to TE_1_	9.39	<1	–	<–15	–	128	–
	TE_0_ to TE_2_	11.27	<1	–	<–15	–	126	–
DBS [24]	TE_1_ to TE_0_	2	<0.6	<2.2	<–20.3	<–16.2	40	40
AC DBS [22]	TE_0_ to TE_1_	4	<1.4	<2.2	<–15	<–10	35	35
	TE_1_ to TE_0_	4	<1.5	<2.2	<–15	<–13	35	35
AGA [17]	TE_0_ to TE_1_	2.2	<3	–	<–10	–	335	–
	TE_0_ to TE_2_	2.2	<3	–	<–10	–	330	–
	TE_0_ to TE_3_	2.2	<3	–	<–10	–	145	–
	TE_2_ to TE_1_	2.2	<3	–	<–10	–	420	–
	TE_2_ to TE_3_	2.2	<3	–	<–10	–	340	–
Dielectric slots [6]	TE_0_ to TE_1_	2.3	<1.2	<1.2	<–16.5	<–6.3	100	50
	TE_0_ to TE_2_	2.4	<0.22	<0.5	<–18	<–9	50	50
Moving asymptotes [33]	TE_0_ to TE_1_	6	<0.1	–	–		60	–
	TE_1_ to TE_2_	6	<0.25	–	–		60	–
This work	TE_0_ to TE_1_	13	<1	<1	<–15	<–15	130	80
	TE_0_ to TE_2_	13	<1	<1	<–15	<–15	100	62
	TE_0_ to TE_3_	13	<1	<1	<–15	<–15	100	70
	TE_1_ to TE_2_	13	<1	<1	<–15	<–15	170	80
	TE_1_ to TE_3_	13	<1	<1	<–15	<–15	65	55
	TE_2_ to TE_3_	13	<1	<1	<–15	<–15	90	75

^a^Sim., simulation; Exp., experimental; AC DBS, axisymmetric constraint direct binary search.

## 4 Conclusions

In conclusion, we have proposed an arbitrary-order mode conversion scheme by optimizing the S-bend structure using the HSO method. Combining PSO with adjoint methods can improve the device’s performance. A group of high-performance MCs including TE_0_ to TE_1_, TE_0_ to TE_2_, TE_0_ to TE_3_, TE_1_ to TE_2_, TE_1_ to TE_3_, and TE_2_ to TE_3_ converters are designed and experimentally demonstrated. The length of all MCs was 13 μm. The measurements show that the operation bandwidths for keeping IL below 1 dB and CT below −15 dB are 80 nm, 62 nm, 70 nm, 80 nm, 55 nm, and 75 nm, respectively, for the TE_0_ to TE_1_, TE_0_ to TE_2_, TE_0_ to TE_3_, TE_1_ to TE_2_, TE_1_ to TE_3_, and TE_2_ to TE_3_ converters. These proposed MCs offer great potential to construct densely integrated and broadband MDM systems for on-chip ultrahigh capacity communications. In addition, our proposed HSO method is highly scalable and can be applied to the optimization of other functional photonic devices on the SOI platform, paving the way for the development and application of integrated nanophotonics.
